# Normative Variability in Retinal Nerve Fiber Layer Thickness: Does It Matter Where the Peaks Are?

**DOI:** 10.1167/tvst.14.5.13

**Published:** 2025-05-12

**Authors:** Sowjanya Gowrisankaran, Ashkan Abbasi, Xubo Song, Joel S. Schuman, Gadi Wollstein, Bhavna J. Antony, Hiroshi Ishikawa

**Affiliations:** 1 Department of Ophthalmology, Casey Eye Institute, Oregon Health & Science University, Portland, OR, USA; 2 Department of Medical Informatics and Clinical Epidemiology, Oregon Health & Science University, Portland, OR, USA; 3 Glaucoma Service, Wills Eye Hospital, Philadelphia, PA, USA; 4 Thomas Jefferson University Sidney Kimmel Medical College, Philadelphia, PA, USA; 5 Vickie and Jack Farber Vision Research Center, Wills Eye Hospital, Philadelphia, PA, USA; 6 Drexel University School of Biomedical Engineering, Science and Health Studies, Philadelphia, PA, USA; 7 Institute of Innovation, Science and Sustainability, Information Technology, Federation University Australia, Ballarat, VIC, Australia

**Keywords:** retinal nerve fiber layer, normative variability, glaucoma, optical coherence tomography

## Abstract

**Purpose:**

Retinal nerve fiber layer thickness (RNFLT), a glaucoma biomarker, has a wide normative range affecting its sensitivity and specificity for abnormality detection. The interindividual RNFLT peak location variability contribution to this wide normative range has not been directly evaluated. The purpose of this study is to assess the effect of RNFLT peak normalization (PN) on normative variability.

**Methods:**

Circumpapillary RNFLT profiles at 1.7 mm radius from the optic nerve head (ONH) were re-sampled from optical coherence tomography (OCT) volumes (Cirrus HD-OCT, 200 × 200) obtained from one eye of 83 healthy individuals. Fovea-ONH axis (FOA) was calculated from corresponding scanning laser ophthalmoscope images. Supratemporal (ST) and infratemporal (IT) RNFLT peaks of each profile were aligned to respective average peak locations. Normative ranges were calculated by averaging individual profiles before and after PN (with and without FOA to horizontal image axis (HA) alignment).

**Results:**

RNFLT-PN resulted in an overall decrease in coefficient of variation (CoV) of the normative range by 4.2% (*P* = 0.02). CoV was reduced by more than 10% in clock-hours 10 (11.9%), 8 (10.6%), 6 (10.4%) after PN, and 7 (16.3%), 10 (11.4%), and 12 (10.4%) after PN with FOA-HA alignment. RNFLT-PN corrected for abnormality categorization because of peak misalignment in RNFLT profiles of healthy and glaucoma suspect subjects.

**Conclusions:**

RNFLT-PN reduces normative variability, especially in the ST and IT regions.

**Translational Relevance:**

RNFLT-PN reduces normative variability and improves sectoral abnormality categorization, potentially leading to better sensitivity and specificity of RNFLT measure in glaucoma detection.

## Introduction

Glaucoma is a slowly progressing disease that leads to irreversible vision loss. Early detection is important to effectively manage this disease and slow its progression. One of the important biomarkers of glaucoma is the retinal nerve fiber layer thickness (RNFLT), which decreases with glaucoma progression. Spectral domain optical coherence tomography (SD-OCT), a widely used technique for structural assessment of the retina and optic nerve head, is used to evaluate RNFLT in glaucoma. In most individuals, RNFLT typically peaks in the supratemporal and infratemporal regions of the retina.^1^ These regions also tend to show changes in RNFLT early in the disease process compared to other retinal regions.^2^ There is, however, large variability across individuals in the thickness amplitude and location of the RNFLT peaks within the superior and inferior regions.^3^^,^^4^

Factors such as age, refractive error, axial length, shape of RNFLT profile have all been shown to contribute to the variability observed in RNFLT measures even within the normal population.^5^^–^^7^ Differences in RNFLT peak locations contribute to the variability in the shape of the RNFLT profile across individuals. Therefore variability in RNFLT peak locations, across visually normal individuals, might also contribute to the wide range and broad peaks in the RNFLT normative data.^3^

The sensitivity and specificity of RNFLT for glaucoma diagnosis could be impacted by this wide normative range.^3^^,^^4^ Additionally, when determining the level of RNFLT abnormality in an individual, their circumpapillary RNFLT profile (typically assessed at a radius of 1.7mm from the optic nerve head (ONH) center) is compared against the normative range obtained from the same circumpapillary radius. Due to inter-individual differences in RNFLT peak locations, it is possible to have misalignment(s) between the location of the individual's peak(s) and the normative peak location(s) leading to false-positive or false-negative categorization of sectoral RNFLT values.

Normalizing RNFLT peak locations across individuals could potentially reduce normative range variability and improve sensitivity and specificity of RNFLT measurements in diagnosing glaucoma. Normalization across individuals is also important for accurate structural assessment and structure-function correlations.

However, the direct contribution of inter-individual differences in RNFLT peak locations to normative variability has not been studied. Hood et al., (2010) utilized major retinal vessel arcade locations to align RNFLT profiles across visually normal individuals. Their results, however, suggest aligning RNFLT profiles at a circumpapillary radius of 1.7mm from the ONH center, based on the retinal vessel arcade location does not significantly reduce normative variability of the RNFLT measure.^8^ Although it is assumed that the variability in RNFLT peak locations significantly contribute to the wide normative range for RNFLT, it is unclear how much reduction in variability is expected if inter-individual differences in RNFLT peak locations are normalized. Additionally, even though there is a close association between retinal blood vessel structure and the nerve fiber bundle, the retinal vessel information that precisely represents the retinal nerve fiber thickness peak locations has not been identified.

To explore the potential of lowering the population variability of the normative RNFLT values, we designed the following two experiments: First, we evaluate the contribution of inter-individual variability in RNFLT peak locations to the normative range variability in RNFLT. Second, we sought to identify the retinal blood vessel feature(s) that best represents RNFLT peak locations in the superior and inferior regions separately.

To evaluate the contribution of RNFLT peak variability to the normative range variability, we normalize RNFLT peak locations across visually normal individuals by directly utilizing individual RNFLT peak locations in the supra- temporal and infra-temporal quadrants. This approach circumvents the need for other features, like the retinal blood vessel features, and enables the direct assessment of the contribution of mismatches in peak locations to the normative range variability.

Although direct utilization of RNFLT peak locations might result in the most straightforward way of normalizing RNFLT peak locations across individuals, we recognize that in patients with glaucoma such peak information might be lacking due to the loss of RNFL. For this reason, alternate stable features which are not affected by the disease process need to be identified for RNFLT normalization to expand its applicability to glaucoma patients when needed. We evaluate the use of retinal vessel location information in the supra-temporal and infra-temporal regions for this purpose. The goal of the study is to gain further insights about the normative RNFLT variability and potentially reduce the variability so that the clinical sensitivity and specificity can be improved.

## Methods

### Subjects and Scans

Optical coherence tomography (OCT) volumes used for this study were from a longitudinal clinical dataset from The University of Pittsburgh that was obtained between 2007 to 2014. Data collection when performed was conducted in accordance with the tenets of the Declaration of Helsinki and the Health Insurance Portability and Accountability Act (HIPPA). The Institutional Review Board of the University of Pittsburgh approved the study, and all subjects gave written consent before participation.

Data from subjects with a visual field mean deviation (MD) of −2 dB or better and no ocular pathologies were used for analysis. OCT scans that were used for analysis had a signal strength of 6 or greater and a corresponding good quality scanning laser ophthalmoscope (SLO) image. 83 OCT volume scans of the ONH region (Cirrus HD-OCT, 200×200, Zeiss, Dublin, CA) of the right or left eyes of 83 healthy individuals were included. All scans were aligned to right-eye laterality.

### Data Pre-Processing

The OCT volume had dimensions of 200×200×1024 pixels along the x, y and z-axis, respectively. For each OCT volume, an enface OCT image was computed by averaging along the depth axis (z-axis). The ONH margin was segmented from the enface image using the ImageJ software,^9^ and the ONH center was obtained from the ONH mask. Centers of all images were aligned to the image center with the x and y coordinates set to 100, using horizontal and/or vertical translations.

**Figure 1.**
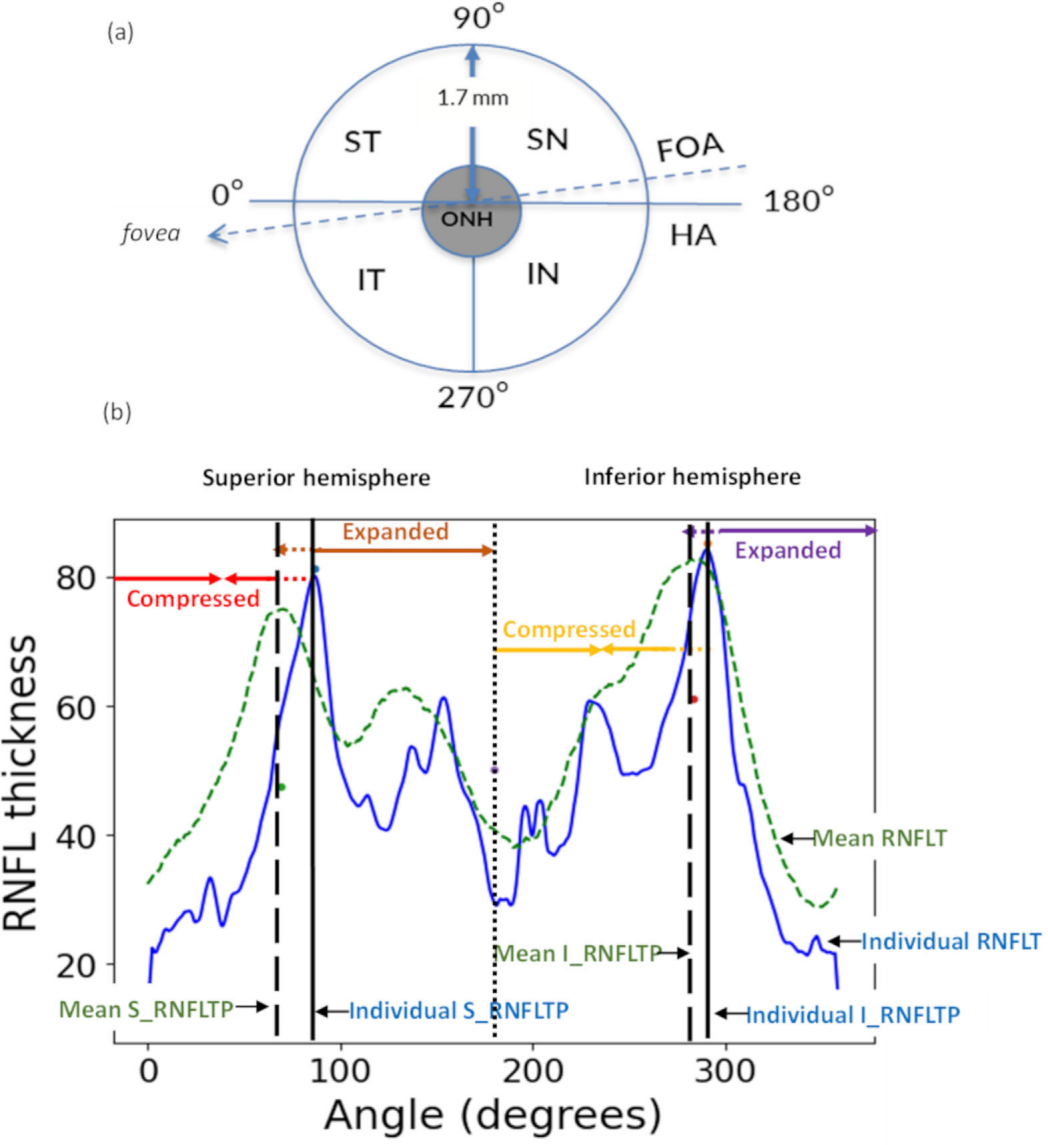
(**a**) Schematic of ONH and surrounding regions (ST, IT, supra-nasal [SN] and infra-nasal [IN]). Hemifields were defined by start and end of the HA and the FOA; (**b**) Alignment of individual RNFLT peak locations (RNFLTP) (*solid vertical black lines*) in the superior (S) and inferior (I) hemispheres (separated by the dotted black line) to the respective mean peak locations (*dashed vertical black lines*). Segments (*solid lines with arrows*) of the individual RNFLT profile (*solid blue*) were interpolated to match the mean RNFLT profile (*dashed green*) segments.

From each volume scan, a 2D RNFLT map was obtained by segmenting the RNFL layer using a custom software.^10^ A circumpapillary RNFLT profile was then re-sampled from this 2D map at 0.5-degree intervals along a circle with a 1.7 mm radius centered on the ONH. This profile was represented in the TSNIT (temporal, superior, nasal, inferior and temporal) format with 0 degrees corresponding to the 9 o'clock hour (Fig. 1a). As the thickness is measured and represented along circumpapillary angular locations around the ONH center, the RNFLT profile is naturally represented in the polar domain. RNFLT peaks were identified on this circumpapillary profile, separately for the superior and inferior retinal regions. The supra-temporal and infra-temporal peaks were defined as the thickest location within 0–110° and 250–360°, respectively. Additionally, the supra-temporal and infra-temporal RNFLT peak locations were also measured at radii of 1.3 mm, 1.5 mm, 1.9 mm and 2.1 mm around the ONH center to evaluate the effect of scan circle diameter on the RNFLT peak locations.

The superior and inferior retinal regions were defined based on two criteria: the horizontal image axis and the fovea to ONH axis (FOA) (Fig. 1a). To determine the FOA, we utilized corresponding SLO images that were obtained concurrently with the OCT volume scans by the native device operating system. The fovea and ONH centers were manually marked on these SLO images, and the angle made by the FOA with the horizontal image axis was calculated. For each image, a rotational correction for this angle was used to align the FOA to the horizontal image axis. The supra-temporal and infra-temporal peak locations were identified for both conditions, with and without alignment of the FOA with the horizontal image axis.

### RNFLT Normative Range and Peak Normalization

The mean peak location across all subjects was calculated separately for the supra-temporal and infra-temporal peaks obtained from the raw RNFLT profiles. The mean and standard deviation of these RNFLT profiles were also calculated to determine the circumpapillary normative range of the RNFLT. We defined the RNFLT values within the 95% confidence interval (CI) as being normal, between 95% and 99% CI as being borderline and below 99% CI as being abnormal.

RNFLT peak normalization was performed in the polar domain by considering the superior and inferior regions, defined by the horizontal image axis, separately. To normalize the peak locations across individuals, the supra-temporal and infra-temporal peak locations from each individual's RNFLT profile were aligned with the respective supra-temporal and infra-temporal mean peak locations across all subjects. This alignment was done by interpolating the portion of the individual's RNFLT profile before and after the peak location, such that the individual's peak location is shifted to match the mean peak location. Depending on whether the individual's peak location is more nasal or temporal in the superior or inferior regions, the RNFLT profile is either compressed or expanded to match the mean peak location. For example, in Figure 1b consider the individual's RNFLT profile (blue line) in the superior region (0° to 180°), the individual's supra-temporal peak (solid black vertical line) is located more nasally compared to the mean peak location (dashed vertical black line) in the mean RNFLT profile (green dashed trace). To align these two peak locations, the portion of the individual's RNFLT profile before the peak location (0° to individual's supra-temporal peak location) was compressed, and the portion after the peak location (individual's supra-temporal peak location to 180°) was expanded to shift the individual's RNFLT peak temporally. Similarly, the individual's infra-temporal peak was aligned to the mean infra-temporal peak location by compressing the individual's RNFLT profile between 180°and individual's infra-temporal peak location, and by expanding the individual's RNFLT profile between individual's infra-temporal peak location and 360°.

After normalizing individual peaks to the respective mean locations, we calculated the normative range again by finding the mean and standard deviation of the RNFLT profiles. The above peak normalization and normative range determination was also repeated after alignment of the FOA to the horizontal image axis.

To determine if RNFLT peak normalization across healthy individuals impacted the normative range variability, we compared coefficient of variation obtained from all clock hour sectors before and after peak normalization, without and with the alignment of FOA to horizontal image axis.

Each clock hour was represented by a 30° sector, and the mean and standard deviation of RNFLT for each of the clock hour sectors across all scans were calculated. The overall coefficient of variation across all clock hours taken together was compared before and after normalization using the Wilcoxon signed rank test for paired samples.

In addition, the clock hour categorization of individual RNFLT profiles as compared to the normative range (calculated without any peak normalization) was evaluated. RNFLT values within the 95% CI was considered normal, between 95% and 99% CI were considered borderline and below 99% CI was considered abnormal. The number of individual profiles of visually normal subjects with at least one clock hour that changed from being abnormal to normal or vice versa following RNFLT peak normalization was calculated. Additionally, a similar analysis of sectoral abnormality categorization was also performed on RNFLT profiles of six patients with a glaucoma suspect diagnosis to demonstrate clinical utility of our method.

### Retinal Vessel Parameters and Its Association With RNFLT Peak Locations

To obtain retinal vessel related parameters, first we segmented retinal vessels from the enface OCT images using deep learning. A UNet architecture^11^ was used with four encoder and four decoder blocks, equipped with skip connections. Each convolution block (filters: 64,128,256 and 512) was made up of two convolution layers, each followed by a batch normalization layer and ReLU activation function. The network was trained on a publicly available retinal fundus image dataset with corresponding annotated retinal vessel masks (CHASE-DB1: retinal vessel reference dataset, 2012, Kingston University Research, Data Repository). These images were converted to grayscale and cropped to match the dimensions of the OCT enface image. During training, we augmented the data using horizontal flips, vertical flips, grid and optical distortions. Augmented data was split at the patient level into training (about 70%) and test sets (about 30%) and performance was evaluated using dice-coefficient. Major retinal vessels on OCT enface images were segmented using this network. Segmented vessels were post-processed using morphological operations to retain thick retinal vessels (defined as greater than 4 pixels) which were then skeletonized. The skeletonized vessel segmentation mask was then represented in the polar domain [polarTransform 2.0 package], with the ONH center as origin and in the TSNIT format that corresponds to the RNFL profile. From this polar representation, the mean angular vessel location at 1.7 mm radius was calculated separately for the supra-temporal and infra-temporal regions.

We also measured the major retinal vessel arcade location for comparison. The location of the major blood vessel arcade, determined based on the most prominent vessel location, within the supra-temporal and infra-temporal regions was manually marked at the 1.7 mm radius around the ONH from the SLO image. Mean major retinal vessel location and location of major retinal vessel arcades were correlated with the corresponding RNFLT peaks and Pearson's correlation coefficients were obtained.

## Results

Table 1 describes the demographic characteristics and relevant clinical information of visually normal subjects whose OCT scans were included in this study. Refractive error information was available for 68 of the subjects. Spherical equivalent refractive error was −1.17 D (±2.4) (mean ± 1 standard deviation (SD)). The visual field mean deviation for the subjects was −0.35 dB ± 0.94 (mean ± SD). The mean (± SD) of the fovea to ONH angle, measured from the corresponding SLO image across all subjects was −10.47° (± 5.44).

**Table 1.** Demographics and Clinical Information of Subjects Included for Normative Range Estimation

	Subjects (n = 83)
Age (y), mean ± SD	53.43 ± 14.91
Gender	
Female	55
Male	28
Race	
White	66
Black	14
Asian	2
Unknown	1
Spherical equivalent refractive error (D), mean ± SD	−1.17 ± 2.38
Visual field mean deviation (dB), mean ± SD	−0.35 ± 0.94

**Figure 2.**
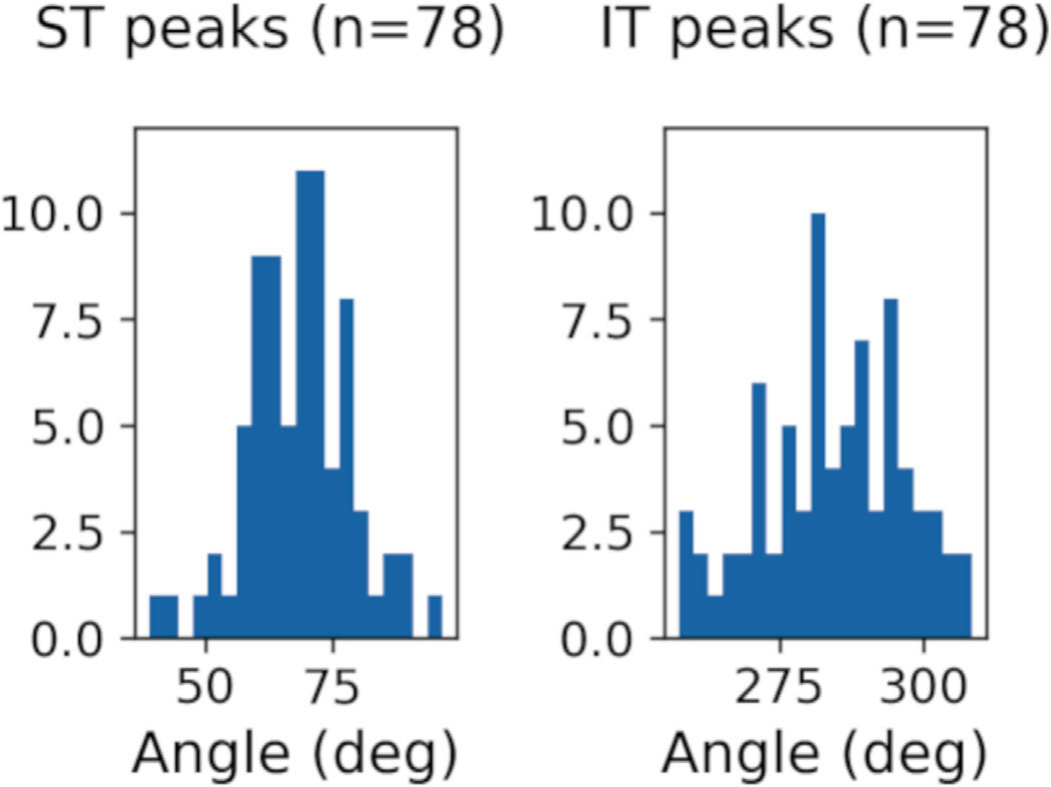
Distribution of ST and IT RNFLT peak angles at 1.7 mm radius from the ONH center.

Of the 83 scans, 78 had circumpapillary RNFL re-sampled scans that were free from noise and artifacts. The distribution of RNFLT peaks across subjects in the supra-temporal and infra-temporal regions (defined based on the image axis) and 1.7 mm radius from the ONH center, is shown in Figure 2. The mean (± SD) of supra-temporal and infra-temporal peaks was 70.5° (± 10.38) and 281.0° (± 12.36), respectively. The RNFLT peaks in the supra-temporal and infra-temporal regions were also measured at four additional scan circle radii (1.3 mm,1.5 mm, 1.9 mm and 2.1 mm). The mean (±SD) of RNFLT peak locations at 1.3 mm, 1.5 mm, 1.9 mm and 2.1 mm were 74.4° (±11.98), 72.3° (±12.01), 65.9° (±11.16), 64.9° (±9.68) for the supra-temporal region and 275.4° (±15.77), 279.7° (±14.55), 288.2° (±11.09), 289.0° (±11.19) for the infra-temporal region. Based on a repeated measures ANOVA analysis, the RNFLT peak locations were significantly different for the different scan circle radii in the supra-temporal (p < 0.001) and the infra-temporal regions (p < 0.001).

### Results of RNFLT Peak Normalization

**Figure 3.**
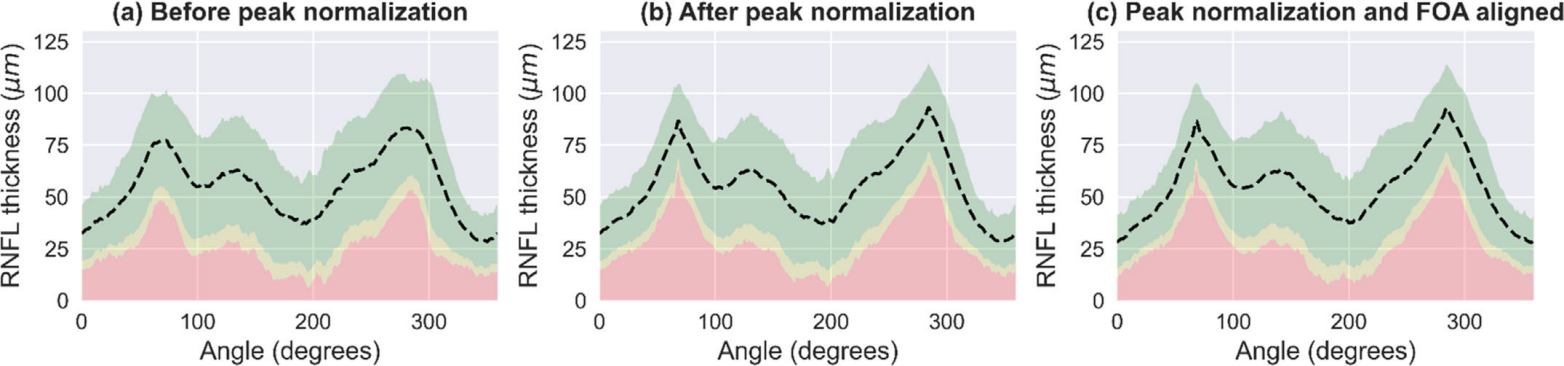
Normative range of RNFLT (**a**) before peak normalization, (**b**) after peak normalization, and (**c**) after peak normalization following rotation of the FOA to align with the horizontal image axis. X-axis is in TSNIT format (0 is CH 9). The dashed line represents mean RNFLT profile. *Green*, within the 95% CI; *yellow*, between 95% and 99% CI; *red*, below 99% CI.

Figure 3a shows the normative range for RNFLT profiles before normalizing the supra-temporal and infra-temporal peaks across individuals. Figure 3b shows the RNFLT profile mean after normalizing individual RNFLT profile peaks in the supra-temporal and infra-temporal regions to the corresponding mean peak locations calculated across all subjects. Figure 3c represents the normative range calculated after peak normalization performed subsequent to rotating the image, to align the FOA to the horizontal image axis. In Figures 3a, 3b and 3c the mean RNFLT measurements along the TSNIT angular locations are represented as the dotted line and regions within the 95% CI is represented by green, between 95% and 99% CI is represented by yellow and below 99% CI is represented by red.

RNFLT was also analyzed by clock hour (CH) regions, RNFLT mean, standard deviation and coefficient of variation for each clock hour was calculated before normalization, after normalization and normalization after FOA alignment (Table 2). There was an overall reduction of 4.2 % in coefficient of variation after peak normalization compared to before normalization (p = 0.02, Wilcoxon-signed rank test) and 4.3% after peak normalization following FOA-horizontal image axis alignment compared to before normalization (p = 0.042). There was no significant difference when comparing coefficient of variation after peak normalization and after peak normalization following FOA-horizontal image axis alignment (p = 0.96). RNFLT peak normalization led to greater reduction in variability around the peak locations in both supra-temporal and infra-temporal region. Coefficient of variation was reduced by about 9 to 10% or more in CHs 6 (10.38%), 8 (10.62%), 10 (11.99%) and 12 (9.42%) after peak normalization. Similar reduction was observed in CHs 7 (16.23%), 10 (11.38%), 12 (10.39%) and 2 (9.96%) after peak normalization following FOA to horizontal image axis alignment.

**Table 2.** Mean (µm), Standard Deviation, and Coefficient of Variation [CoV] (%) for RNFLT Measures Calculated Based on Clock Hour Sectors Before and After Peak Normalizations

Clock Hour	RNFLT Before Peak Normalization [CoV]	RNFLT After Peak Normalization [CoV]	RNFLT After Peak Normalization + FOA-ONH Alignment [CoV]
1	59.27 ± 13.13 [22.16]	59.13 ± 13.17 [22.28]	57.42 ± 12.81 [22.31]
2	55.14 ± 14.23 [25.80]	54.07 ± 14.46 [26.74]	58.84 ± 13.67 [23.23]
3	40.21 ± 11.61 [28.87]	40.80 ± 11.88 [29.12]	43.98 ± 13.43 [30.54]
4	45.48 ± 14.12 [31.03]	46.26 ± 13.87 [29.97]	42.01 ± 12.43 [29.57]
5	62.89 ± 13.41 [21.33]	61.40 ± 13.01 [21.29]	58.86 ± 13.00 [22.08]
6	78.65 ± 14.47 [18.40]	80.14 ± 13.21 [16.49]	78.74 ± 13.63 [17.31]
7	70.33 ± 17.76 [25.26]	70.77 ± 17.27 [24.20]	74.73 ± 15.81 [21.16]
8	36.60 ± 10.37 [28.33]	36.12 ± 9.15 [25.32]	41.62 ± 11.47 [27.57]
9	32.90 ± 7.78 [23.65]	32.91 ± 7.72 [23.44]	30.50 ± 6.7 [21.96]
10	45.78 ± 10.46 [22.84]	45.42 ± 9.13 [20.10]	42.59 ± 8.62 [20.24]
11	70.57 ± 14.10 [19.99]	72.85 ± 13.51 [18.54]	71.14 ± 14.6 [20.52]
12	62.66 ± 14.71 [23.47]	62.29 ± 13.24 [21.26]	64.12 ± 13.48 [21.03]

**Figure 4.**
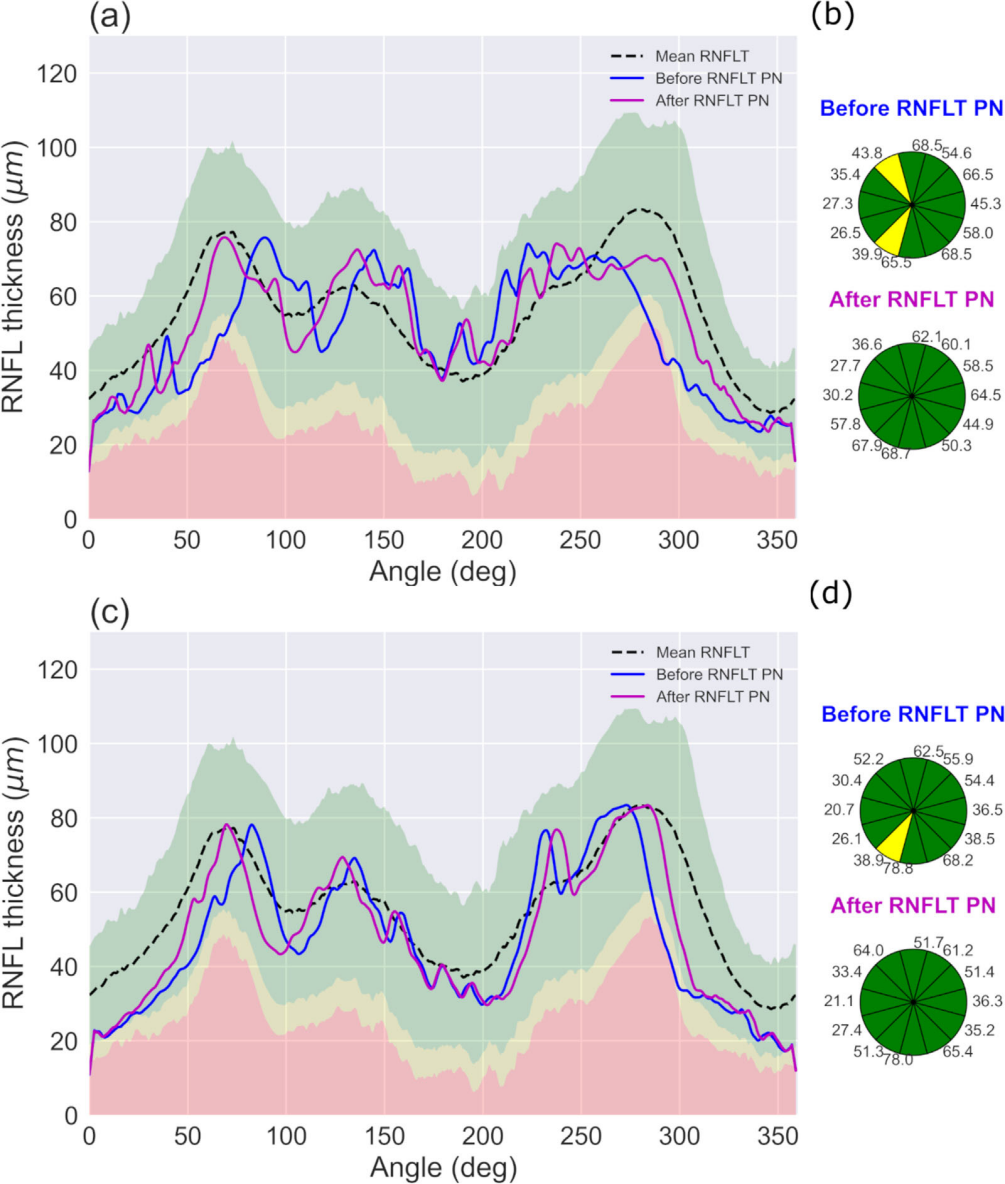
(**a**) and (**b**) RNFLT profiles of visually normal subjects before (*blue*) and after (*magenta*) peak normalization relative to normative range and their corresponding clock hour (*CH*) plots. Numbers adjacent to the sectors represent RNFLT (in µm) for that sector.

In our dataset of normal eyes, six of the 78 scans had at least one clock hour with RNFLT values that was identified as being borderline, totally eight sectors had borderline values. Following peak normalization, the clock hour defects were no longer present in four of the eight sectors in four of the six scans. Figure 4 shows the RNFLT profile of two subjects, with supra-temporal and infra-temporal peaks misaligned with respective mean peak locations. Applying our peak normalization method improves alignment of RNFLT peaks and therefore, in scans with abnormality mis-categorization due to peak misalignment, improves relative categorization of clock hour defects.

**Figure 5.**
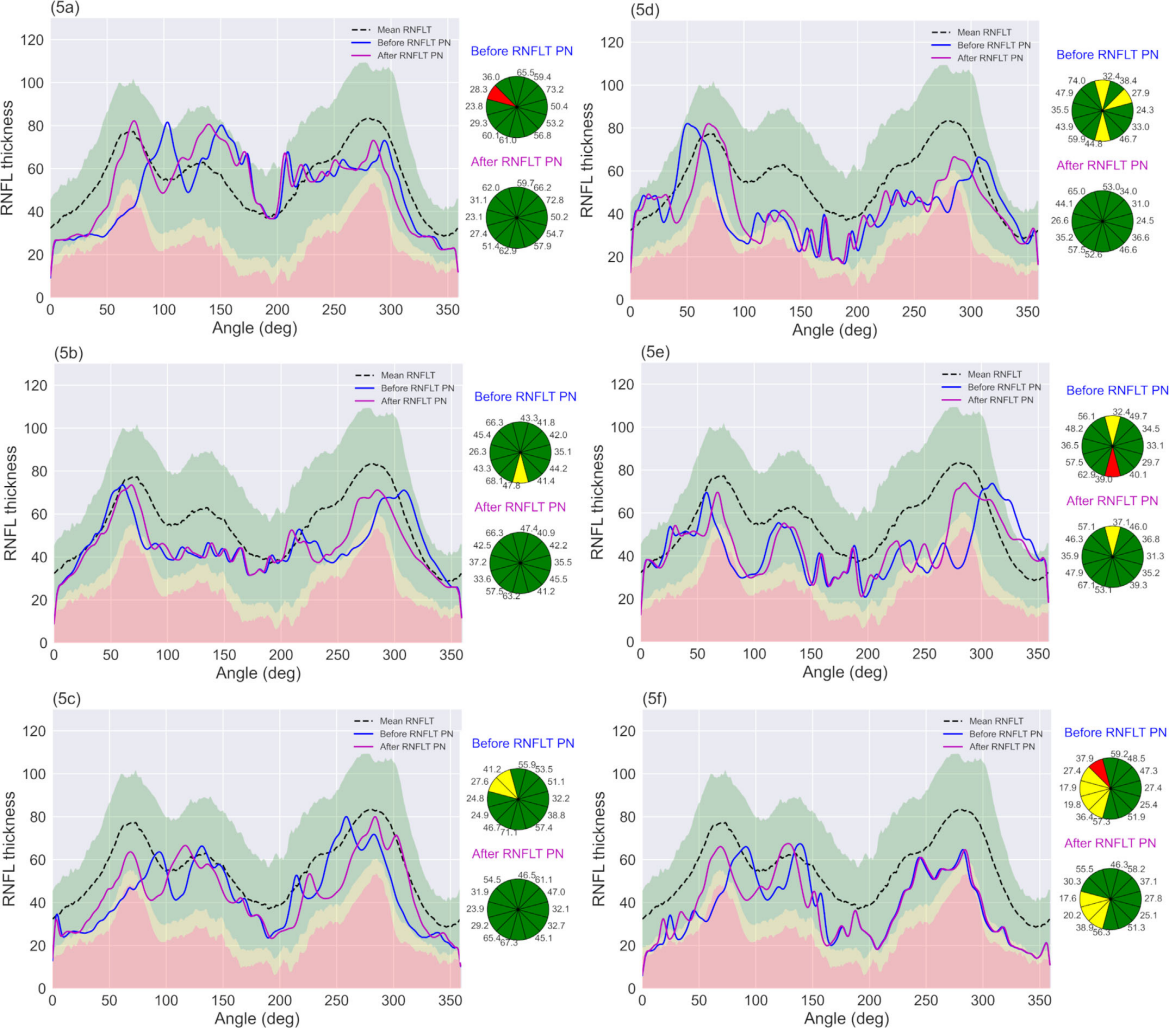
(**a**–**f**) RNFLT profiles of glaucoma suspects before (*blue*) and after (*magenta*) peak normalization relative to normative range and their corresponding clock hour plots.

To demonstrate the clinical utility of the RNFLT peak normalization approach, we applied the method to six glaucoma suspect cases (average visual field mean deviation of −2.43 dB (±1.8)). Figure 5a–f shows the RNFLT profiles before (blue) and after (magenta) peak normalization relative to the normative range, and their corresponding clock hour plots. As shown in the figure, our method corrects for all sectoral misclassifications that result from misaligned peaks (Fig. 5a–f). But the method does not change the categorization when there is no peak misalignment as seen in the inferior region of Figure 5f, where the borderline RNFLT abnormality remains after peak normalization, but the mis-categorization in the superior region is corrected.

### Association of Blood Vessel Location Parameters and RNFLT Peaks

**Figure 6.**
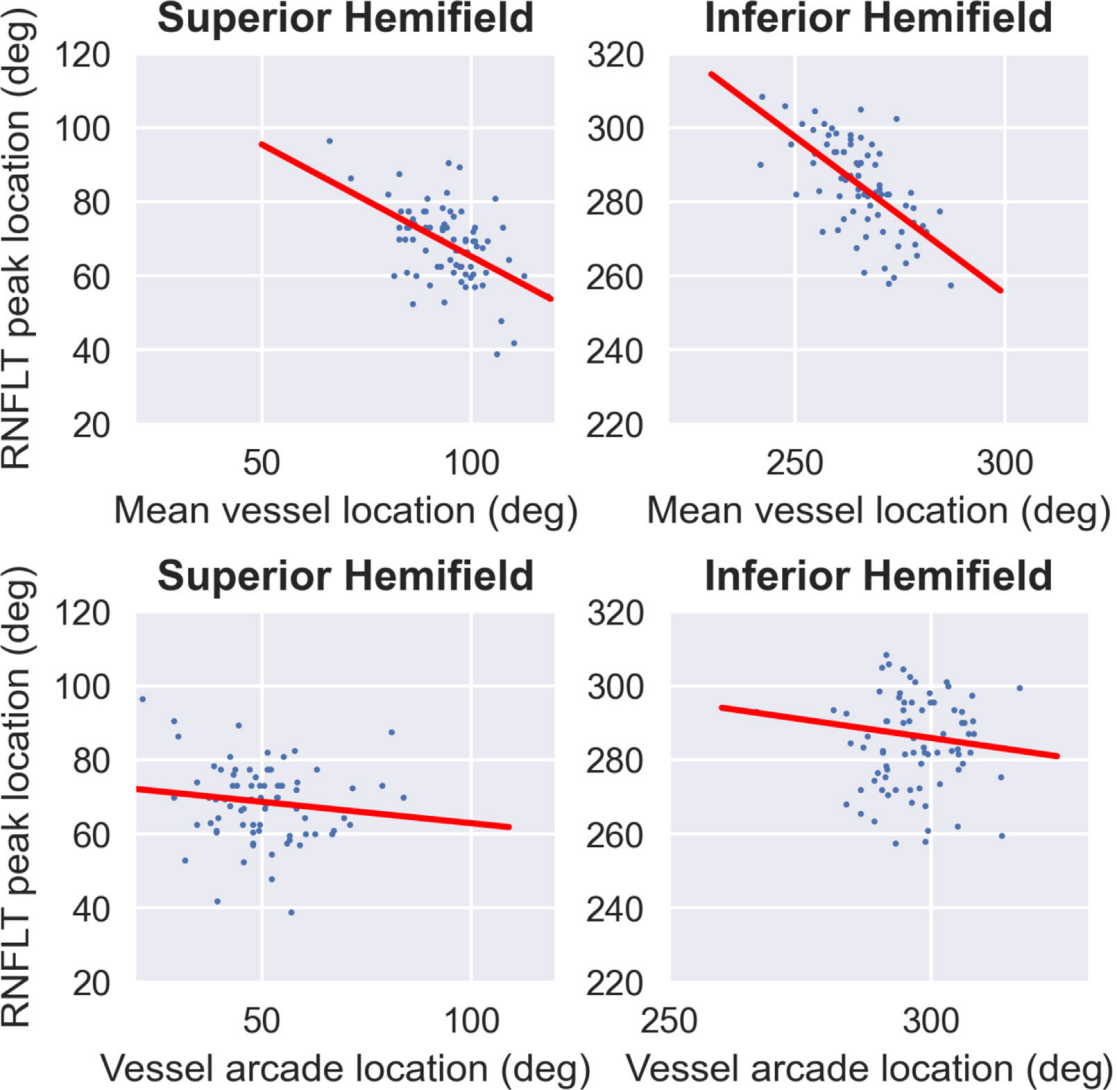
Correlations between RNFLT peak locations and blood vessel parameters: mean blood vessel location (*top*), major blood vessel arcade location (*bottom*).

Pearson's correlation coefficient between the supra-temporal and infra-temporal RNFLT peak locations and the corresponding blood vessel mean locations were −0.52 (p < 0.001) and −0.62 (p < 0.001), respectively. The correlation coefficient for the two RNFLT peak locations and the corresponding superior and inferior major retinal vessel arcade locations were −0.13 (p = 0.25) and 0.004 (p = 0.96) for the supra-temporal and infra-temporal regions, respectively (Fig. 6). Correlations between the FOA and the supra-temporal and infra-temporal RNFLT peak locations were 0.34 (p = 0.002) and 0.14 (p = 0.2), respectively. Aligning FOA to the image axis did not improve correlations between RNFLT peak locations and blood vessel location measures.

## Discussion

In this study we evaluated the contribution of RNFLT peak locations to the normative range variability of RNFLT measures from visually normal individuals. Normalizing the location of RNFLT peaks separately for the superior and inferior regions, across individuals, resulted in statistically significant reduction in normative range variability. Greater than 10% reduction in coefficient of variation was observed for clock hours 6,8 and 10 after peak normalization and in clock hours 7, 10 and 12 after peak normalization following FOA alignment. Our peak normalization approach has, for the first time, shown a reduction in normative range of RNFLT, albeit localized. Changes within these quadrants are particularly critical for glaucoma diagnosis, as early changes in RNFLT are often observed within these regions.^4^

The localized reduction in normative range limits, could be due to the selection of just two locations for normalization. As can be observed in the individual RNFL profiles, shape variability of the RNFLT profiles is not limited to the major peak locations. Some individuals have multiple peaks in the supra-temporal region with varying amplitudes, and additional peak locations in the supra-nasal and infra-nasal regions. However, the presence of supra-nasal and infra-nasal peaks is not consistent across individuals and therefore was not considered for normalization in this study. It is possible that a similar normalization approach when considering multiple peak locations might result in further reduction in normative range variability around the regions considered.

Different FOA angles might also add to the variability observed in RNFLT profiles across individuals. Aligning the FOA with the horizontal image axis, should account for the variability in RNFL peak location due to differences in FOA, which defines the anatomical separation between the superior and inferior retinal regions. Our results for the range of FOA angle observed are similar to those reported previously.^12^ By rotating the images based on the FOA angle, we normalize its contribution prior to evaluating the additional variability due to RNFLT distribution across the retina. By performing this alignment, a further reduction in RNFLT coefficient of variation was observed in clock hours 2, 7 and 9. However, there was no significant correlation between FOA angle and RNFLT peak locations, and correcting for the FOA did not improve correlations between retinal vessel mean location and RNFLT peak locations.

It is possible that the variability in axial length across subjects leading to different scan circle locations around the ONH, resulting from magnification, could also contribute to the normative variability after peak alignment.^13^^,^^14^ In this study, axial length information was not available for all patients, therefore the magnification of scan circle related to the axial length was not taken into account. Previous work by Cheol YY (2011) showed that there was a moderate correlation between axial length and the location of RNFLT peaks.^15^ Our assessment of scan circle radius on RNFLT peak location also showed that the scan circle radii had a significant effect on the RNFLT peak locations. However, the expected effect of magnification due to the axial length variability is relatively small because circular resampling with 1.7mm radius is going through approximately the center location of the RNFLT plateau area.^16^ Finally, our entry criteria also limited the refractive errors of the subjects in our study, minimizing the variability and therefore impact of axial length on our results. Although lack of axial length information is not ideal, practically it does not introduce significant limitations for our study.

Contribution of other factors such as age, gender, refractive error and, retinal blood vessels related information, on RNFLT variability have been previously evaluated, but were not considered in this study.^17^^–^^21^ It is possible that considering these factors in addition to peak location could further reduce normative variability limits.

Previous report that aligned temporal vessel arcade locations by shifting them, did not show any effects on the RNFLT normative range. Hood et al.^8^ used temporal vessel arcade location (which they defined as the mean location between the major temporal vein and the artery) of individuals along the 3.4 mm diameter circle to normalize RNFLT profiles across these individuals. They reported a correlation of 0.72 between the superior temporal vessel arcade location and the superior temporal RNFLT profile edge, and a correlation of 0.34 for the inferior temporal vessel arcade location and the inferior temporal RNFLT profile edge. However, they concluded that aligning temporal vessel arcade locations did not significantly reduce RNFLT normative variability. Another study showed an overall reduction of about 4% to 10% in coefficient of variation of RNFLT profile, in healthy individuals from different races, after compensating for vessel density along with other demographic factors such as race and age.^22^ In our study we show that directly aligning the RNFLT peaks (measured from RNFLT profile), leads to an overall reduction in coefficient of variation of about 4% when considering all clock hour sectors. And, a greater reduction in coefficient of variation was obtained around the supra-temporal and infra-temporal peak locations. Peak normalization resulted in a maximum of about 12% reduction in coefficient of variation for the 10 o’clock sector and peak normalization after accounting for the FOA resulted in a maximum reduction of about 16% along the 7 o’clock sector. We also showed that there is a stronger correlation between mean retinal vessel location in the supratemporal and infratemporal region and the respective RNFLT peak locations, compared to the major temporal vessel arcade location in our study. Variability in the temporal arcade location across individuals, the method used to determine the vessel location information, variability in determining the ONH center and the scatter in the correlation between vessel locations and RNFLT peaks could all contribute to the discrepancies between our findings and previous results.

We recognize that our approach relies on detecting RNFLT peak locations for peak normalization. In patients with glaucoma, the RNFLT peak locations may be difficult to detect or absent due to the loss of the RNFL. Although peak alignment with the normative range might not be critical for long term follow up of glaucoma patients, initial assessment of severity of RNFLT reduction could still be affected by peak misalignments and could be particularly important in patients who are closer to the lower amplitude limit of the normative range.

In this study we evaluated the association of retinal vessels mean location with RNFLT peak locations to explore its utility as a surrogate for RNFLT peak location for normalization. We show that there is a reasonable correlation between mean retinal vessel location and RNFLT peak locations in both the supra-temporal and infra-temporal regions. However, it is possible that even though there is a reasonable correlation, this single vessel related parameter might be insufficient to obtain accurate estimates of the RNFLT peak locations. Therefore further investigation is required to determine whether the retinal blood vessel-based features, or other disease invariant OCT features, could be beneficial in accurately predicting RNFLT peak locations for peak alignment, even in the presence of glaucomatous changes.

Another strength of our method is its ability to reduce false-positive errors caused by peak misalignment between individual RNFLT profiles and the normative data. In our sample cohort of normal subjects, the proportion of individuals with large deviations from mean RNFL peak locations was low. Therefore, to demonstrate our method's potential clinical application, we applied peak normalization to six subjects with a glaucoma suspects diagnosis. These subjects had near normal visual field mean deviations and showed abnormalities in sectoral RNFLT in the clock hour plots prior to peak normalization. The presence of sectoral misclassification at first presentation might influence clinical decision making, therefore applying peak normalization to improve accuracy of sectoral abnormality classification could be clinically useful in patients with a glaucoma suspect diagnosis. For the six glaucoma suspect cases presented, peak normalization corrects all sectoral misclassifications arising from peak misalignment. These results point to the potential clinical application of our approach in improving accuracy of sectoral classification at first presentation, especially in patients with a glaucoma suspect diagnosis. A potential way to adopt our approach in the clinical setting would be to incorporate peak normalization in the OCT device output, so that the clinician is provided with peak normalized RNFLT profiles and their corresponding sectoral abnormality categorization.

Our method of peak normalization will also be beneficial in populations with greater number of temporally or nasally displaced peaks, like in myopic eyes.^15^ Additionally, the RNFLT profiles closer to the lower bound of the normative range are more prone to sectoral misclassifications due to peak misalignments with the normative range. Our approach effectively aligns the peaks of the individual RNFLT profiles to the normative mean, even in the absence of refractive error or axial length information, which could be beneficial in reducing false positives in myopic patients. This approach could also be useful in demographics with significantly different distributions of supra-temporal and infra-temporal peak locations compared to the normative range that they are compared to for diagnostic purposes, especially when normative data with a similar demographic is unavailable or hard to obtain.

## Conclusions

RNFLT peak normalization has region specific effects on reducing variability in the normative range, with greater reduction in the superior-temporal and inferior- temporal regions. Our approach reduces false-positive errors that arise due to peak misalignments between individual RNFL profiles and the normative data range.

## References

[1] Gabriele ML, Ishikawa H, Wollstein G, et al. Peripapillary nerve fiber layer thickness profile determined with high speed, ultrahigh resolution optical coherence tomography high-density scanning. *Invest Ophthalmol Vis Sci*. 2007; 48: 3154–3160. 17591885 10.1167/iovs.06-1416 PMC1950319

[2] Hood DC, Wang DL, Raza AS, Gustavo de Moraes C, Liebmann JM, Ritch R. The locations of circumpapillary glaucomatous defects seen on frequency-domain OCT scans. *Invest Ophthalmol Vis Sci*. 2013; 54: 7338–7343. 24135758 10.1167/iovs.13-12680 PMC3823545

[3] Hong SW, Ahn MD, Kang SH, Im SK. Analysis of peripapillary retinal nerve fiber distribution in normal young adults. *Invest Ophthalmol Vis Sci*. 2010; 51: 3515–3523. 20164448 10.1167/iovs.09-4888

[4] Hood DC. Improving our understanding, and detection, of glaucomatous damage: an approach based upon optical coherence tomography (OCT). *Prog Retin Eye Res*. 2017; 57: 46–75. 28012881 10.1016/j.preteyeres.2016.12.002 PMC5350042

[5] Alasil T, Wang K, Keane PA., et al. Analysis of normal retinal nerve fiber layer thickness by age, sex, and race using spectral domain optical coherence tomography. *J Glaucoma*. 2013; 22: 532–541. 22549477 10.1097/IJG.0b013e318255bb4a

[6] Knight OJ, Girkin CA, Budenz DL, et al. Effect of race, age, and axial length on optic nerve head parameters and retinal nerve fiber layer thickness measured by Cirrus HD-OCT. *Arch Ophthalmol*. 2012; 130: 312–318. 22411660 10.1001/archopthalmol.2011.1576 PMC5536837

[7] Yamashita T, Asaoka R, Tanaka M, et al. Relationship between position of peak retinal nerve fiber layer thickness and retinal arteries on sectoral retinal nerve fiber layer thickness. *Invest Ophthalmol Vis Sci*. 2013; 54: 5481–5488. 23847316 10.1167/iovs.12-11008

[8] Hood DC, Salant JA, Arthur SN, Ritch R, Liebmann JM. The location of the inferior and superior temporal blood vessels and interindividual variability of the retinal nerve fiber layer thickness. *J Glaucoma*. 2010; 19: 158–166. 19661824 10.1097/IJG.0b013e3181af31ec PMC2889235

[9] Schneider C, Rasband W, Eliceiri K. NIH Image to ImageJ: 25 years of image analysis. *Nat Methods*. 2012; 9: 671–675. 22930834 10.1038/nmeth.2089 PMC5554542

[10] Ishikawa H, Stein DM, Wollstein G, et al. Macular segmentation with optical coherence tomography. *Invest Ophthalmol Vis Sci*. 2005; 46: 2012–2017. 15914617 10.1167/iovs.04-0335 PMC1939723

[11] Ronneberger O, Fischer P, Brox T. U-Net: Convolutional networks for biomedical image segmentation. Navab N, Hornegger J, Wells W, Frangi A, eds. Medical image computing and computer-assisted intervention – MICCAI 2015. Lecture Notes in Computer Science (vol 9351). Berlin: Springer, 2015: 234–241.

[12] Chauhan BC, Danthurebandara VM, Sharpe GP, et al. Bruch's membrane opening minimum rim width and retinal nerve fiber layer thickness in a normal white population. A multicenter study. *Ophthalmology*. 2015; 122: 1786–1794. 26198806 10.1016/j.ophtha.2015.06.001 PMC4698808

[13] Savini G, Barboni P, Parisi V, Carbonelli M. The influence of axial length on retinal nerve fibre layer thickness and optic-disc size measurements by spectral-domain OCT. *Br J Ophthalmol*. 2012; 96: 57–61. 21349942 10.1136/bjo.2010.196782

[14] Öner V, Özgür G, Türkyilmaz K, Şekeryapan B, Durmus M. Effect of axial length on retinal nerve fiber layer thickness in children. *Eur J Ophthalmol*. 2014; 24: 265–272. 23918073 10.5301/ejo.5000345

[15] Yoo YC, Lee CM, Park JH. Changes in peripapillary retinal nerve fiber layer distribution by axial length. *Optom Vis Sci*. 2012; 89: 4–11. 21983121 10.1097/OPX.0b013e3182358008

[16] Gabriele ML, Ishikawa H, Wollstein G, et al. Optical coherence tomography scan circle location and mean retinal nerve fiber layer measurement variability. *Invest Ophthalmol Vis Sci*. 2008; 49: 2315–2321. 18515577 10.1167/iovs.07-0873 PMC2728289

[17] Budenz DL, Anderson DR, Varma R, et al. Determinants of normal retinal nerve fiber layer thickness measured by stratus OCT. *Ophthalmology*. 2007; 114: 1046–1052. 17210181 10.1016/j.ophtha.2006.08.046 PMC2916163

[18] Yamashita T, Sakamoto T, Yoshihara N, et al. Correlations between retinal nerve fiber layer thickness and axial length, peripapillary retinal tilt, optic disc size, and retinal artery position in healthy eyes. *J Glaucoma*. 2017; 26: 34–40. 27753756 10.1097/IJG.0000000000000550

[19] Wagner FM, Hoffmann EM, Nickels S, et al. Peripapillary retinal nerve fiber layer profile in relation to refractive error and axial length: Results from the Gutenberg health study. *Transl Vis Sci Technol*. 2020; 9(9): 35. 10.1167/tvst.9.9.35 PMC7445357 32884859

[20] Qiu K, Schiefer J, Nevalainen J, Schiefer U, Jansonius NM. Influence of the retinal blood vessel topography on the variability of the retinal nerve fiber bundle trajectories in the human retina. *Invest Ophthalmol Vis Sci*. 2015; 56: 6320–6325. 26436884 10.1167/iovs.15-17450

[21] Arnould L, Guillemin M, Seydou A, et al. (2020) Association between the retinal vascular network and retinal nerve fiber layer in the elderly: The Montrachet study. *PLoS One*. 2020; 15(10): e0241055. 33085730 10.1371/journal.pone.0241055 PMC7577490

[22] Chua J, Schwarzhans F, Nguyen DQ, et al. Compensation of retinal nerve fibre layer thickness as assessed using optical coherence tomography based on anatomical confounders. *Br J Ophthalmol*. 2020; 104: 282–290. 31118184 10.1136/bjophthalmol-2019-314086 PMC7025730

